# Effectiveness of the Combination of Enalapril and Nifedipine for the Treatment of Hypertension versus Empirical Treatment in Primary Care Patients

**DOI:** 10.3390/jcdd10060243

**Published:** 2023-05-31

**Authors:** Humberto Badillo-Alonso, Marisol Martínez-Alanis, Ramiro Sánchez-Huesca, Abel Lerma, Claudia Lerma

**Affiliations:** 1 Centro de Investigación en Ciencias de la Salud (CICSA), FCS, Universidad Anáhuac México Campus Norte, Huixquilucan Edo. de Mexico 52786, Mexico; humbertobadilloalonso@gmail.com; 2 Jalalpa el Grande Health Center, Mexico City Health Secreatariat, Mexico City 01377, Mexico; 3 School of Engineering, Universidad Anahuac Mexico, Huixquilucan 52786, Mexico; marisol.martinez2@anahuac.mx; 4 Instituto Nacional de Cardiologia Ignacio Chávez, Mexico City 04480, Mexico; farmacologia.medica@gmail.com; 5 Institute of Health Sciences, Universidad Autónoma del Estado de Hidalgo, San Agustín Tlaxiaca 42160, Mexico; abel_lerma@uaeh.edu.mx

**Keywords:** hypertension control, primary care, clinical trial

## Abstract

Hypertension in Mexico has a prevalence of 32% and is the second most widespread cause of consultation in primary care. Only 40% of patients in treatment have a blood pressure (BP) below 140/90 mmHg. This clinical trial aimed to compare the effectiveness of the combination of enalapril and nifedipine versus the empirical treatment for hypertension in patients with uncontrolled BP in a primary care center in Mexico City. Participants were randomized to treatment with enalapril and nifedipine (combination group) or to continue with the empirical treatment. Outcome variables were BP control, therapeutic adherence, and adverse effects at 6 months of follow-up. At the end of the follow-up period, BP control (64% versus 77%) and therapeutic adherence (53% versus 93%) showed an improvement from the baseline values in the group that received the combination treatment. BP control (51% versus 47%) and therapeutic adherence (64% versus 59%) in the group who received the empirical treatment did not show improvement from the baseline to follow-up. Combined treatment was 31% more efficacious than conventional empirical treatment (odds ratio = 3.9), which yielded an incremental clinical utility of 18% with high tolerability extent among patients in primary care in Mexico City. These results contribute to the control of arterial hypertension.

## 1. Introduction

Hypertension is a disease that contributes most to all-cause morbidity and mortality worldwide [1,2]. Hypertension can be detected in the community and in primary care settings, and several effective medications are available at low cost to treat patients with hypertension and reduce the risk of sequalae. Improving effective treatment coverage for patients with hypertension is a goal of many global, regional, and national initiatives and programs [3]. The care of hypertension, including detection, treatment, and management, varies substantially around the world and even within the same region of the world. In Mexico, the National Health and Nutrition Survey 2020 (ENSANUT 2020) reports that the prevalence of hypertension in Mexican adults was 49.4%, using the American Heart Association (AHA) classification as a reference. Among these adults, 70% were diagnosed with hypertension at the time of the survey. According to the JNC-7 classification (used in ENSANUT 2020), only 30.2% of Mexican adults had hypertension and 51% of them were unaware of having this disease.

Hypertension can be detected at primary care and low-cost treatments can effectively control it. Although lifestyle modification (nonpharmacological treatment) is important, it has been very difficult to apply it at the individual and population level, and is often not sufficient by itself to control blood pressure. Therefore, effective pharmacological management is essential for controlling hypertension. However, with the increasing number and diversity of pharmacological agents available that encompasses several key and complementary drug classes, treatment options are now complex and need to be simplified [4]. Furthermore, other barriers to an effective antihypertensive treatment involve the healthcare providers, who may lack a complete understanding of the appropriate use of the different pharmacological classes and individual agents, be reluctant to use standardized treatment algorithms, and are driven by “clinical or therapeutic inertia” (the phenomenon of not initiating therapy immediately), which delays dose increases or the addition of other pharmacological agents when indicated. At the primary care level, factors, such as the lack of accessibility to health centers and clinics, the limited availability of affordable and reliable drugs, the inability to maintain follow-up and treatment programs once initiated, and budgetary constraints preventing the widespread use of antihypertensive drugs, all contribute to low rates of effective treatment [5]. In Mexico, the treatment of arterial hypertension is regulated by the Mexican Official Standard NOM_ 030-SSA2 for the prevention, treatment and control of arterial hypertension and clinical practice guidelines. However, in many cases, patients receive the empirical treatment, which is defined as “the treatment of diseases by means whose usefulness has been demonstrated by the experience of the primary care physician”, without strictly following the current clinical practice guidelines, and includes four main classes of antihypertensive drugs: angiotensin-converting enzyme inhibitors, angiotensin receptor blockers, calcium channel blockers, and thiazide and thiazide-like diuretics. These drugs are generally prescribed in monotherapy. Any of these four classes of antihypertensive drugs can be used as initial treatment unless there are specific contraindications. However, to achieve blood pressure control (systolic blood pressure < 140 mmHg and diastolic blood pressure < 90 mmHg), effective hypertension treatment usually requires at least two antihypertensive medications from different complementary classes [6,7,8,9,10].

When combining antihypertensive drugs, the aim should be to maximize the effects with a decrease in adverse reactions. The combination chosen for this study is an angiotensin-converting enzyme inhibitor “enalapril” and a calcium channel blocker “nifedipine” which has an antihypertensive effect and the potential to mitigate side effects of the substances given separately. The combination based on enalapril and nifedipine is justified on several pharmacological, therapeutic, and clinical grounds [11]. This combination therapy is metabolically neutral and has been shown to offer consistent advantages in relation to new-onset diabetes mellitus when compared to other classical combination therapy, such as beta-blockers and thiazide diuretics [12]. This combination also presents an important clinical advantage in terms of tolerability, as it favors a significant reduction in the adverse effects of one component (ankle edema favored by calcium channel blockers) through the antagonistic peripheral vascular actions of angiotensin-converting enzyme inhibitors [13]. Moreover, the fixed-dose combination of angiotensin-converting enzyme inhibitors and calcium channel blockers may offer an important additional advantage in relation to patient compliance when compared to the separate administration of the two drugs, while maintaining blood pressure control and renal and cardiovascular protection efficacy [14]. Both enalapril and nifedipine are antihypertensive drugs that are included in the basic drug list at primary care and are in continuous supply due to their low cost in Mexico, representing an early antihypertensive response because it is made up of first-line drugs that simplify the treatment regimen with the benefit of contributing to therapeutic adherence.

The combination of enalapril and nifedipine has not been studied in relation to a treatment scheme, which is highly variable and is based on the experience of the primary care physician (empirical treatment). Although the effectiveness of drug combinations for the management of arterial hypertension has been demonstrated in several studies, the specific circumstances at primary care have not been evaluated. Prior to this clinical trial, no direct comparison studies between empirical treatment and this fixed-dose combination at primary care have been conducted that would demonstrate if the combination of enalapril and nifedipine is superior to non-fixed free combinations. The aim of this work was to compare the effectiveness of the combination of enalapril and nifedipine for the treatment of hypertension versus empirical treatment, with respect to blood pressure control, therapeutic adherence, and adverse effects, in patients with uncontrolled blood pressure at primary care.

## 2. Materials and Methods

### 2.1. Study Design and Patients

The study design was a randomized clinical trial. It was an experimental study comparing two groups with the aim to evaluate the antihypertensive effectivity of a combined treatment, while describing therapeutic adherence and any possible adverse effects. The combined treatment consisted of enalapril (one tablet of 10 mg every 12 h) and nifedipine (one tablet of 10 mg every 12 h).

The participants included in the study were selected from a group of patients with a hypertension diagnosis that attended “Dr. Manuel Escontria” Health Center in Mexico City. All participants agreed to take part in the study by signing an informed consent. Participants were selected in a non-probabilistic manner with consecutive cases and random assignment to the intervention groups. This was a prospective, randomized, open, blinded-endpoint study. Inclusion criteria considered patients, both male and female, over 40 years of age who had a systolic blood pressure between 140 and 180 mmHg and a diastolic blood pressure between 90 and 110 mmHg. All patients had to be recently diagnosed with hypertension or based on a medical opinion, must benefit from a change in treatment.

Exclusion criteria considered any patient who had suffered an acute myocardial infarction in prior months, who had arrhythmias, unstable angina, heart failure, diagnosis of a cerebrovascular accident or renal failure. For fertile women, anyone taking contraception or pregnant were excluded, because oral contraceptives can prevent an effective antihypertensive treatment in certain patients [15]. Additionally, any patient taking an immunosuppressive treatment, with known hypersensitivity to calcium channel blockers or angiotensin converting enzyme inhibitors, were excluded from our study.

Any patient who voluntarily decided to leave, abandoned the study, or presented adverse effects that could put their health at risk, was eliminated.

This study was approved by the Bioethics Committee of our institution (protocol number 201626). It was carried out in accordance with the provisions contained in the General Health Law of Mexico and the ethical principles contained in the Declaration of Helsinki.

### 2.2. Sample Size and Sampling Method

The sample size was calculated considering the proportion of patients who are expected to experience a reduction in their blood pressure after application of a combined antihypertensive therapy treatment (40%) and the proportion of patients whose blood pressure figures will be reduced by continuing with an empirical treatment (25%). The sample size was calculated using the 2-proportion comparison formula for experimental studies (Equation (1)).

(1) $$n={\left[\frac{z_{\alpha }\sqrt{2p\left(1-p\right)}+z_{\beta }\sqrt{p_{1}\left(1-p_{1}\right)+p_{2}\left(1-p_{2}\right)}}{p_{1}-p_{2}}\right]}^{2}$$

A confidence level of 95% and a power of 80% were considered. The calculated minimum sample size was 145.86, rounded to 146 subjects for both the experimental group and the non-experimental group. Though only 292 patients were required, a total of 328 participated in this study.

The sampling process was carried out by a group of 12 primary care physicians and each of them selected a minimum of 30 patients via consecutive sampling. Allocation of the patients to each intervention group was randomized.

### 2.3. Study Protocol

Participating patients signed an informed consent after one of the physicians explained the purpose of this study. They could be either newly diagnosed as hypertensive or those who, in the opinion of their doctor, could benefit from a change in treatment based on the proposed combined therapy.

**Figure 1:**
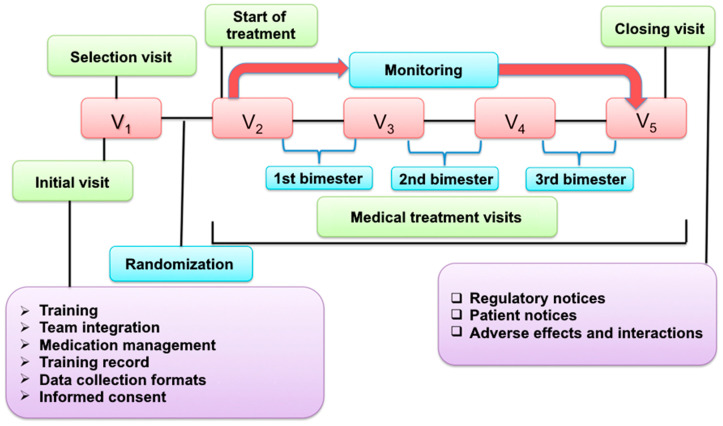
Flow chart of the study protocol.

Patient monitoring lasted 6 months, during which 5 control visits were carried out (Figure 1). In the initial visit (V1), training was conducted and recorded, the medical team was formed, and they were given instructions on the management of the medication. The data collection formats were tested, and patients were selected and randomly assigned to the two intervention groups. The following visits were carried out every two months. In the first bimester visit (V2), the pharmacological treatment was started in the intervention groups. The next two visits (V3 and V4) were the follow-up of the treatment, and the final visit (V5) conducted was the last follow-up and closure of our study.

During each visit, blood pressure, weight and height were recorded; and laboratory tests, including glycemia, glycated hemoglobin, total cholesterol, triglycerides, creatinine, and uric acid were conducted. At the start and the end of the study, systematic analytical controls were registered. The possibility of presenting adverse effects and their degree were recorded whenever they presented. Blood pressure was measured in the morning, before taking the medication, after resting for 5 min, and in the dominant arm. Two measurements were performed 2 min apart in a sitting position, and the average of both was recorded. In each consultation, the results of the Morisky–Green–Levine test for therapeutic adherence and notification of suspected adverse drug reactions were applied and recorded in the patient’s medical record.

#### 2.3.1. Assessment of Blood Pressure, Vital Signs, Anthropometric Variables, and Biochemical Variables

All blood pressure measurements were conducted using an automatic and validated electronic device (Omron M4). All anthropometric measurements were performed using a scale with a validated digital stadiometer (Seca 213). All laboratory samples were processed in the laboratory of the health center using automated equipment (Cobas c11).

#### 2.3.2. Assessment of Therapeutic Adherence and Adverse Reactions

The Morisky–Green–Levine test consists of four questions where it is specified, according to the value of the answers obtained, if the patient has had therapeutic adherence to a pharmacological treatment or if the adherence is not adequate. In all cases, the questions must be answered with a “yes” or a “no”. Adherents (ADT) are those who answer NO to the four questions and non-adherents (NADT) are those who answer YES to one or more questions. The test has shown a good correlation between adherence and blood pressure control [16].

In the event of any adverse reaction, the observation was conducted during the clinical interview using the Suspected Adverse Reactions Report format from the National Center for Pharmacovigilance in Mexico.

#### 2.3.3. Study Variables

Systolic blood pressure and diastolic blood pressure were measured in mmHg as described above. The main outcome was controlled blood pressure (yes or no), defined as systolic blood pressure < 140 mmHg and diastolic blood pressure < 90 mmHg. Adherence to treatment and adverse reactions were the secondary outcomes. The intervention variable was the antihypertensive treatment (combined or empirical treatment).

Anthropometric variables were age (years), sex (female or male), body weight (kg), height (cm), and body mass index (kg/m^2^). Clinical variables included comorbidities (diabetes, obesity, metabolic syndrome, and dyslipidemia). Blood chemistry laboratory variables included glucose (mg/dL), glycated hemoglobin (%), uric acid (mg/dL), creatinine (mg/dL), total cholesterol (mg/dL), and triglycerides (mg/dL).

### 2.4. Statistical Analysis

Most continuous variables did not have a normal distribution (Kolmogorov–Smirnov test with *p* < 0.05). Therefore, these variables were reported as median (25th percentile—75th percentile) and were compared between treatments using the Mann–Whitney U test. Within each treatment group, the medians evaluated at 2, 4, and 6 months were compared against the evaluation baseline using the Wilcoxon test. The study variables were compared with respect to uncontrolled hypertension (controlled vs. uncontrolled) using the Mann–Whitney U test (ordinal variables) or Chi-square test (nominal variables). To evaluate the efficacy of the combined treatment, we calculated several indices. For each treatment, we assessed the incidence of controlled blood pressure (number of patients with controlled blood pressure/total number of patients who received the treatment), expressed as percentage. Then, we calculated the clinical utility as the difference between incidence in the combined treatment—incidence in the empirical treatment (expressed as percentage), which corresponds to the absolute risk reduction (ARR), expressed as a proportion. The relative risk (RR) was calculated as incidence of combined treatment/incidence of empirical treatment. The number required to be treated (NRT) was calculated as 100%/clinical utility. Statistical analysis was performed using SPSS Statistics 21.0 (IBM Corp., Armonk, NY, USA) and Microsoft Excel 2017.

## 3. Results

### 3.1. Baseline Characteristics

The characteristics of the study participants are shown in Table 1. Both treatment groups were similar, except for a lower proportion of overweight or obese patients in the group with combined treatment compared to the group with empirical treatment.

Table 2 shows a detailed description of the prescribed drugs in both the study groups. Compared to the group with empirical treatment, the group with combined treatment had more patients with prescribed oral hypoglycemic drugs (glibenclamide), less patients with other antihypertensive drugs (captopril, metoprolol, telmisartan and losartan), and less patients with diuretic prescription (hydrochlorothiazide, and chlorthalidone). There were no significant differences in other prescribed drugs.

### 3.2. Outcome Variables and Treatment Efficacy

Table 3 shows the results of the outcome variables. At baseline, compared to the empiric treatment group, the group with the combined treatment had a slightly larger proportion of patients with controlled blood pressure (51 vs. 64%), less treatment adherence (64 vs. 53%), and less adverse reaction (2 vs. 1%). However, after 6 months of treatment, compared to the empiric treatment group, the group with the combined treatment had a notably larger proportion of patients with controlled blood pressure (47 vs. 77%), more treatment adherence (59 vs. 93%), and less adverse reaction (2 vs. 1%).

**Figure 2:**
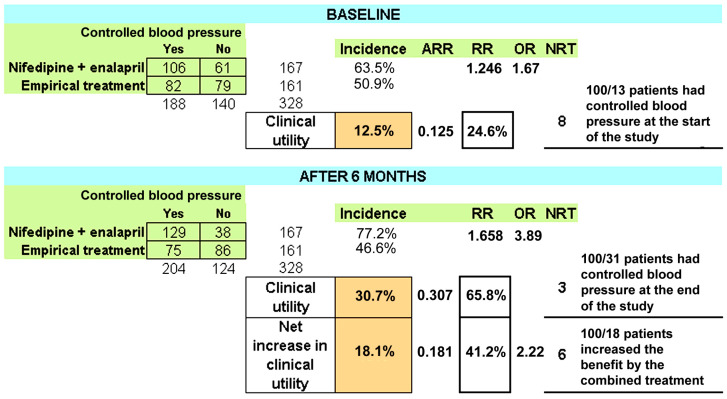
Assessment of treatment efficacy for blood pressure control with the combined treatment (nifedipine + enalapril) versus the empirical treatment. BP = blood pressure, ARR = absolute risk reduction, RR = relative risk, OR = odds ratio, NRT = number required to be treated.

Figure 2 shows the estimation procedure of treatment efficacy for hypertension control of the combined treatment (enalapril plus nifedipine) versus the empirical treatment group. At baseline, the combined treatment was 12.5% superior to the empirical treatment (63.5–50.9%), which corresponds to an absolute risk reduction ARR = 0.125, with a relative risk (63.5/50.9) = 1.246, and an odds ratio = ((106 × 79)/(82 × 61) = 1.67). After 6 months of treatment, the combined treatment was 30.7% superior (77.2–46.6%), which corresponds to an absolute risk reduction of 0.307, a relative risk = 77.2%/46.6% = 0.307, and an odds ratio = ((129 × 86)/75 × 38) = 3.89). The relative risk (of controlling blood pressure) increased from 1.246 to 1.658 after 6 months of combined treatment, raising the clinical protective effect by 41.2% (24% to 64%), leading to a net increase in clinical utility of 18.1% (12.5% to 30.7%). This clinical utility indicates that the number of patients required to be treated (NRT) to control blood pressure decreased from eight to three patients.

### 3.3. Anthropometric, Blood Pressure and Laboratory Variables Follow-Up

**Figure 3:**
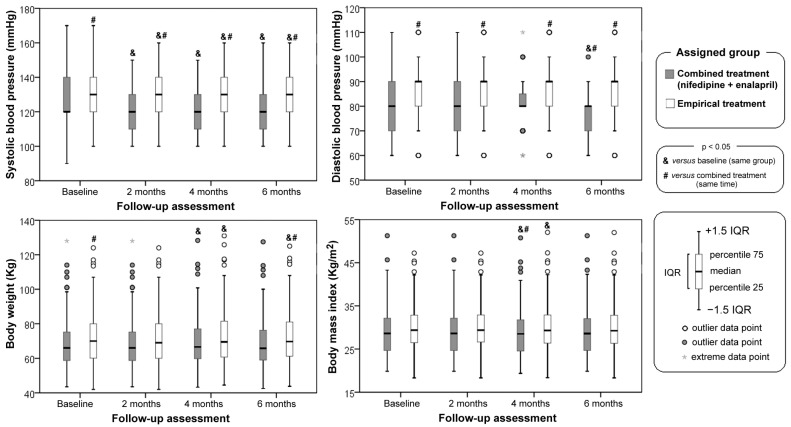
Baseline and follow-up assessment of blood pressure and anthropometry. IQR = inter-quartile range.

Figure 3 shows that systolic blood pressure was higher in the empirical treatment group compared to the combined treatment group at all assessment times (including baseline). In both groups, the antihypertensive treatment decreased the systolic blood pressure at all times of follow-up compared to baseline.

Diastolic blood pressure was larger in the empirical treatment group compared to the combined treatment group at all assessment times (Figure 3). The combined treatment group had a significant decrease from the baseline at all the follow-up months. The combined treatment group had the largest decrease with respect to baseline after six months, while the empirical group remained unchanged compared at the same time.

Body weight was larger in the empirical treatment group compared to the combined treatment throughout the study (Figure 3). Compared to baseline, changes in body weight occurred in both groups after four months of follow-up, and the increase remained in the empirical treatment group.

Regarding body mass index (Figure 3), compared to baseline, significant changes presented after four months of follow-up in both the groups; the empirical treatment group only presented changes at four months in relation to the combined treatment group at the same time.

**Figure 4:**
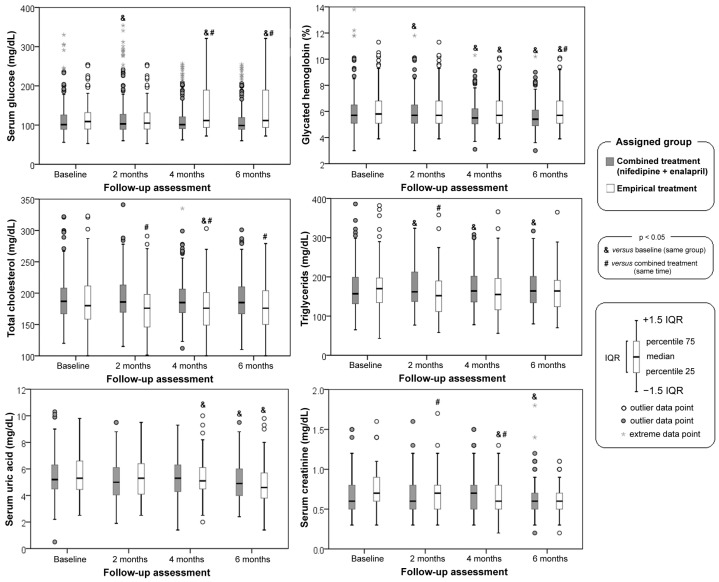
Baseline and follow-up assessment of serum glucose, glycated hemoglobin, total cholesterol, triglycerides, serum uric acid, and serum creatinine. IQR = inter-quartile range.

Figure 4 shows the assessment of serum glucose, glycated hemoglobin, total choles-sterol, triglycerides, uric acid, and creatinine. Glucose did not show significant changes in the combined treatment group during the study, but for the empirical treatment group, an increase was observed at 4 and 6 months of months of treatment. Glycosylated hemoglobin showed changes in the combined treatment group at 2, 4 and 6 months compared to the empirical treatment group. The combined treatment group presented changes at 2, 4 and 6 months in relation to the baseline of the same group, while the empirical treatment group showed changes at 4 and 6 months compared to baseline in the same group. Cholesterol was higher in the combined treatment group and showed no changes during the study. The empirical treatment group showed a slight decrease at 2, 4 and 6 months in relation to the combined treatment at the same time. Triglycerides showed a slight increase at 2, 4 and 6 months in the combined treatment group compared to baseline, while in the empirical treatment group changes were only recorded at 2 months with a slight decrease as compared to the combined treatment. At 6 months of evaluation, the combined treatment group had a slight decrease in uric acid levels in reference to the baseline of the same group, while the empirical treatment showed a decrease at 4 and 6 months with respect to the baseline of the same group. Serum creatinine in the empirical treatment group showed a decrease at 2 and 4 months with respect to the combined treatment group and a decrease at 6 months in the combined treatment group with respect to the beginning of the study.

## 4. Discussion

The main aim of this work was to assess the efficacy of an intervention to improve the arterial blood pressure control in primary care patients diagnosed with systemic arterial hypertension by initiating (or changing) treatment to a fixed combination of enalapril and nifedipine and comparing it with the empirical treatment. The results show that after 6 months of treatment, the combination of enalapril and nifedipine improved the control of arterial blood pressure compared with the conventional empirical treatment. The combined therapy also improved the adherence without adverse effects. An important contribution is that this research was performed in the primary care setting, which is one of the largest settings that treats most of the population but has low rates of controlled blood pressure. In regions such as Latin America, low blood pressure control rates are a significant public health issue, considering the high hypertension prevalence, which is about 30 to 45% in the general population and this tend to increase rapidly with age [17].

For a long time, clinicians have been overconfident regarding monotherapy. Guidelines from the European Society for Hypertension (ESH), European Society of Cardiology (ESC) and the Joint National Committee 8 (JNC 8) from the United States of America indicate that most patients will need two or more medications to control arterial blood pressure and shed light on recommendations about possible combinations [18]. Moreover, it is recommended to reduce the number of pharmaceutical dosage forms throughout the combination of fixed doses [19]. A combined therapy is recommended to patients with high cardiovascular risk and subclinical organic damage, as well as those irresponsive to monotherapy [20]. Several clinical trials have documented that the physician’s decision is one of the main reasons contributing to the lack of update in the antihypertensive treatment and thus in the control of blood pressure [21,22,23].

The combined therapy is aimed to provide a synergistic effect, more tolerability, higher patient’s therapeutic adherence [24], simplify the treatment, improve the blood pressure control, and reduce the cardiovascular morbidity and mortality. Nowadays, double or triple drug combinations are available to hypertensive patients with good clinical results, that is, adequate therapeutic adherence and low profile of adverse effects [25]. The administration of an efficacious treatment is key to reducing the risk of other diseases related to systemic arterial hypertension, such as myocardial infarction and cerebrovascular event [26]. Despite these well-established concepts, systemic arterial hypertension is still treated inadequately around the world [27].

This study also contributes to a change in the way general practitioners prescribe medications, since it is common to find physicians who are reluctant to modify or increase the antihypertensive treatment in patients who present non-controlled blood pressure [28]. Therefore, to overcome this reluctance in upgrading medication, it is important for physicians and healthcare providers to become conscious about the elevated risk in those patients who do not reach the minimum goal of blood pressure within the first year of treatment. The use of educational programs also has an important role in enhancing the conduct of physicians during systemic arterial hypertension treatments [29].

The lack of adherence to antihypertensive therapy is probably the most important reason for non-controlled arterial blood pressure and it is influenced by multiple interrelated factors [30]. To understand the lack of therapeutic adherence and its associated factors, it is important to determine correct intervention strategies. There are several factors related to poor control of arterial blood pressure, for example, those related to patients or the role of the health system [29]. This study has shown that the combination of enalapril and nifedipine from the beginning of the treatment favors therapeutic adherence.

Follow-up laboratory tests were performed on all patients who participated in this study to describe the biochemical changes during the course of the combined therapy. Although the laboratory results were statistically significant for some analytes, these did not result in a change of the clinical state of the patients and the variations were minimal. Further studies are required to test if the combination of enalapril and nifedipine, as a treatment for systemic arterial hypertension, has any beneficial effects in other metabolic variables usually monitored by the blood biochemical tests in primary care.

It seems that drug combinations, not restricted to hypertensive drugs but also those with statins and antidiabetics, will be widely used in chronic and degenerative diseases in the near future [25]. Combined antihypertensive therapies are more effective, better tolerated, safer and has less economic impact than monotherapy [31], however, there is a lack of evidence to guide the selection of drugs under consideration of different populations, according to age, sex, ethnic group, and comorbidities. We hypothesize that the assessed drug combination may prevent complications in the long term.

A double-blind study was not performed, since no placebo was used and patients with arterial hypertension with different degrees of evolution and clinical complications were included, thereby making it necessary to test the intervention with groups with greater control and stratification to standardize the treatment. Furthermore, although the allocation of patients was randomized, the fact that the selection of patients could be decided based on medical opinion increased the risk of bias in the selection process. We do not know the impact of this intervention on other clinical aspects, such as biochemical parameters, adherence to treatment of diabetes and other comorbidities, survival or risk of hospitalization; these factors should be explored in future research.

## 5. Conclusions

The combination of enalapril and nifedipine for the treatment of hypertension is more effective in controlling blood pressure than the empirical treatment in patients with uncontrolled blood pressure in primary care. After six months of treatment, the combination showed an increase of 41.2% in the clinical protector effect and an increase of 18.1% in net clinical utility Treatment adherence also improved in the patients undergoing the combined treatment, without any difference in adverse reactions. These findings support the use of simple therapeutic schemes for an easier, more accessible, and effective treatment of hypertension in primary care patients.

## Figures and Tables

**Table 1:** Sociodemographic and clinical characteristics of the study participants at baseline. Data are shown as median (percentile 25—percentile 75) or absolute value (percentage).

Variable	Empirical Treatment (N = 161)	Combined Treatment (Enalapril + Nifedipine) (N = 167)	*p* Value
Age (years)	61 (53–70)	62 (55–71)	0.501
BMI (kg/m^2^)	29.2 (26.3–32.8)	28.6 (24.7–32.0)	0.092
Female sex	120 (75%)	123 (74%)	0.855
Overweight or obese	139 (86%)	122 (73%)	0.003
Diabetes mellitus	91 (56%)	94 (65%)	0.966
Dyslipidemia	58 (36%)	69 (41%)	0.325
Metabolic syndrome	72 (45%)	75 (45%)	0.972
Uncontrolled diabetes	43 (47%)	34 (36%)	0.126
Uncontrolled dyslipidemia	58 (36%)	69 (41%)	0.325
Uncontrolled metabolic syndrome	74 (45%)	75 (45%)	0.972

**Table 2:** Prescribed drugs during the study. Data are shown as absolute value (percentage).

Variable	Empirical Treatment	Enalapril + Nifedipine	*p* Value
Acetylsalicylic acid	19 (12%)	16 (10%)	0.515
Metformin	74 (45%)	91 (54%)	0.098
Glibenclamide	20 (12%)	42 (25%)	0.003
Linagliptin	1 (1%)	1 (1%)	0.742
Acarbose	3 (2%)	3 (2%)	0.640
Fast insulin	2 (1%)	4 (2%)	0.360
Glargine insulin	39 (24%)	29 (17%)	0.126
NPH insulin	1 (1%)	2 (1%)	0.514
Captopril	49 (30%)	0 (0%)	<0.001
Hydrochlorothiazide	6 (4%)	0 (0%)	0.013
Chlorthalidone	4 (3%)	0 (0%)	0.057
Metoprolol	26 (16%)	0 (0%)	<0.001
Propranolol	2 (1%)	1 (1%)	0.486
Telmisartan	12 (7%)	0 (0%)	<0.001
Losartan	44 (27%)	0 (0%)	<0.001
Alopurinol	0 (0%)	2 (1%)	0.258
Pravastatin	33 (21%)	34 (20%)	0.542
Atorvastatin	5 (3%)	6 (4%)	0.525
Bezafibrate	17 (11%)	25 (15%)	0.232
Verapamil	1 (1%)	0 (0%)	0.491
Furosemide	1 (1%)	0 (0%)	0.491

**Table 3:** Outcome variables. Data are shown as absolute value (percentage).

**Baseline**	**Empirical Treatment**	**Enalapril + Nifedipine**	***p* Value**
Controlled blood pressure			0.022
Yes	82 (51%)	106 (64%)	
No	79 (49%)	61 (36%)	
Treatment adherence			0.038
Yes	103 (64%)	88 (53%)	
No	58 (36%)	79 (74%)	
Adverse reactions			0.298
Yes	3 (2%)	1 (1%)	
No	158 (98%)	166 (99%)	
**After 6 months of treatment**	**Empirical treatment**	**Enalapril + nifedipine**	***p* value**
Controlled blood pressure			<0.001
Yes	75 (47%)	129 (77%)	
No	86 (53%)	38 (23%)	
Treatment adherence			<0.001
Yes	95 (59%)	155 (93%)	
No	66 (41%)	12 (7%)	
Adverse reactions			0.298
Yes	3 (2%)	1 (1%)	
No	158 (98%)	166 (99%)	

## Data Availability

The data presented in this study are available on request from the corresponding author.
